# Natural history predicts patterns of thermal vulnerability in amphibians from the Atlantic Rainforest of Brazil

**DOI:** 10.1002/ece3.7961

**Published:** 2021-11-19

**Authors:** Leildo M. Carilo Filho, Bruno T. de Carvalho, Bruna K. A. Azevedo, Luis M. Gutiérrez‐Pesquera, Caio V. Mira‐Mendes, Mirco Solé, Victor G. D. Orrico

**Affiliations:** ^1^ Programa de Pós‐Graduação em Zoologia Universidade Estadual de Santa Cruz Ilhéus Brasil; ^2^ Departamento de Ciências Biológicas Universidade Estadual de Santa Cruz Ilhéus Brasil; ^3^ Department of Evolutionary Biology (EBD) Spanish National Research Council Madrid Spain; ^4^ Programa de Pós‐Graduação em Sistemas Aquáticos Tropicais Universidade Estadual de Santa Cruz Ilhéus Brasil; ^5^ Herpetology Section Zoologisches Forschungsmuseum Alexander Koenig Bonn Germany

**Keywords:** climate changes, CT_Max_, deforestation, future vulnerability, tropical amphibians, warming tolerance

## Abstract

In the Brazilian Atlantic Rainforest (AF), amphibians (625 species) face habitat degradation leading to stressful thermal conditions that constrain animal activity (e.g., foraging and reproduction). Data on thermal ecology for these species are still scarce. We tested the hypothesis that environmental occupation affects the thermal tolerance of amphibian species more than their phylogenetic relationships. We evaluated patterns of thermal tolerance of 47 amphibian species by assessing critical thermal maxima and warming tolerances, relating these variables with ecological covariates (e.g., adult macro‐ and microhabitat and site of larval development). We used mean and maximum environmental temperature, ecological covariates, and morphological measurements in the phylogenetic generalized least squares model selection to evaluate which traits better predict thermal tolerance. We did not recover phylogenetic signal under a Brownian model; our results point to a strong association between critical thermal maxima and habitat and development site. Forest species were less tolerant to warm temperatures than open area or generalist species. Species with larvae that develop in lentic environment were more tolerant than those in lotic ones. Thus, species inhabiting forest microclimates are more vulnerable to the synergistic effect of habitat loss and climate change. We use radar charts as a quick evaluation tool for thermal risk diagnoses using aspects of natural history as axes.

## Graphical abstract

This study describes a strong association between critical thermal maximums and the habitat and place of development for 47 species of amphibians from the Atlantic Forest. We show that ecological profiles and aspects of natural history (rather than phylogenetic relationship) are the main predictors of thermal sensitivity.

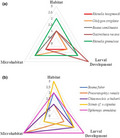

**Research Highlights**

- The thermal vulnerability among the Atlantic Rainforest amphibians is correlated to ecological pattern of habitat occupancy.
- Forest species that reproduce in streams and whose adults are arboreal are the most susceptible to climate change.

## 1 INTRODUCTION

Among vertebrates, amphibians have particular characteristics (e.g., permeable skin) that make them extremely dependent on abiotic factors such as temperature and humidity (Duellman & Trueb, 1994). Amphibians are the most endangered group of vertebrates in the world (Alroy, 2015; Ceballos et al., 2017; Stuart et al., 2004). Recent studies estimate that approximately 200 amphibian species (2.4% of all global diversity) are already extinct, and direct and indirect factors related to human activities threaten 41% (Alroy, 2015; Hoffmann et al., 2010; IUCN, 2020). Even those classified as Least Concern (LC) by the IUCN (International Union for Conservation of Nature—an international effort to estimate the extinction risk of species) may be facing declines and/or loss of populations (Ceballos et al., 2017). With ca. 1,137 species, Brazil is the country with the largest amphibian richness (Frost, 2020; Segalla et al., 2019), but a significant number of these species are still classified as Data Deficient (approx. 281 spp.) (IUCN, 2021; Tapley et al., 2018).

The Brazilian Atlantic Forest is one of Earth's biodiversity hot spots, having been reduced to only 12.4% of its original area (SOS Atlantic Forest, 2017). It harbors more than 50% (ca. 650 spp.) of amphibian species recognized for Brazil (Myers et al., 2000; Rossa‐Feres et al., 2017; Segalla et al., 2019). In the south of Bahia state, the Atlantic Rainforest is particularly important for amphibian conservation given the high number of species, several endemic (e.g., Dias et al., 2014; Mira‐Mendes et al., 2018). The overall diversity of amphibians in this region remains understudied given the numerous recent additions to knowledge of regional diversity by descriptions of new species (Carnaval et al., 2009; Dias et al., 2017, 2020; Orrico et al., 2018). Therefore, southern Bahia can be a considerate priority area for amphibian conservation taking into account the high diversity of species, lineages, distinct life history guilds, and current pressures as climate change and fragmentation (Campos et al., 2017, 2020; Loyola et al., 2008; Vasconcelos et al., 2014).

Both on a local (southern Bahia) and on a biome (Brazilian Atlantic Forest) scale, habitat fragmentation and climate may act in synergism producing disconnection between the habitat from different amphibian ontogenetic stages (Becker et al., 2007). Therefore, amphibian conservation efforts can benefit from considering life history variation in habitat use, because microhabitat, activity windows, and sensibility may vary along with the ontogenetic development in amphibians (Enriquez‐Urzelai et al., 2019).

Thermal exposure is among the main threats to amphibian conservation in the Atlantic Forest (Hof et al., 2011; Silvano & Segalla, 2005). Therefore, it is paramount to investigate ecophysiological aspects linked to the thermal niche (e.g., CT_Max_) to understand the consequences of climate change for these animals (Li et al., 2013). In a warming Earth, ecophysiological studies provide data to assess climatic vulnerability of species, mainly in ectotherm animals such as amphibians (Tejedo et al., 2012). The critical thermal maximum (CT_Max_) is the thermal point where the animal loses its locomotor ability to respond to a stressful thermal environment (Cowles & Bogert, 1944; Taylor et al., 2020). The use of CT_Max_ combined with the environmental thermal data allows to infer the species level of sensitivity (e.g., warming tolerance) to future global warming scenarios (Deutsch et al., 2008; Duarte et al., 2012; Gutiérrez‐Pesquera et al., 2016; Simon et al., 2015). Additionally, studies have recovered phylogenetic signals in thermal niche dimensions (e.g., CT_Max_ and CT_Min_) for amphibians (Gutiérrez‐Pesquera et al., 2016; Hof et al., 2010). The presence of phylogenetic signal has been tested in tadpoles of anuran species from the Atlantic Rainforest of southern Bahia, but nothing is known about adults (Gutiérrez‐Pesquera et al., 2016). Since the larval and adult stages are exposed to different environmental conditions, it is paramount to understand the physiological dimensions of both development stages and elucidate any phylogenetic signatures involved, and the implications for the species fitness (i.e., population growth rate).

In Brazil, research on the impacts of global warming on amphibians is still scarce (Winter et al., 2016). Herein, we evaluate patterns of upper thermal tolerance by accessing the CT_Max_ in adults of 47 amphibian species from the Atlantic Forest of southern Bahia. We expect that macro‐ and microhabitat play a role in buffering against the exposition to high temperatures. Furthermore, we investigate patterns of thermal tolerance among species groups organized by macro‐ and microhabitats of adults, and site of larval development. We hypothesize that forest‐associated species have lower heat tolerance than generalists and those who inhabit open areas. We also expect that arboreal species will reveal lower CT_Max_ values if compared with terrestrial, fossorial, and cryptozoic species. We used a phylogenetic model to identify whether ecological, allometric, and microclimatic covariates can predict thermal tolerance of species. Therefore, we use model selection to evaluate whether the CT_Max_ of the studied species (obtained from experiments with adults) results from an evolutionary process predicted by Brownian motion, as already predicted for CT_Max_ of larval forms of species in the study region (Gutiérrez‐Pesquera et al., 2016). Last, we use radar charts as a rapid assessment tool for thermal risk diagnoses, using CTMax as a ranking factor to categorize ecological groups according to their thermal sensitivity.

## 2 MATERIALS AND METHODS

### 2.1 Specimen collection and study area

We captured specimens from Atlantic Forest fragments in southern Bahia state, Brazil. The climate of the region is tropical humid, Af (tropical wet), and Am (tropical monsoon) in the Köppen classification, with annual average temperature and precipitation around 25°C and 1200 mm, respectively. Sampling was performed at the following locations: Almadina municipality (14°42′0.51″S, 39°37′48″W), Serra Bonita Private Reserve of Natural Heritage (15º23′S, 39º33′W, Camacã municipality), Provisão Farm (14°39′19.09″S, 39°13′13.90″W, Ilhéus municipality), cabruca agroforestry system in Universidade Estadual de Santa Cruz campus (14°47′45″S, 39°10′20″W, Ilhéus municipality), Michelin Ecological Reserve (Igrapiúna municipality), Bonfim Farm (14°36′24.68″S, 39°21′17.99″W, Uruçuca municipality), village of Acuípe (Ilhéus–Una Road, Ilhéus municipality), and Camamú Bay Islands (Maraú municipality). We performed visual encounter surveys through habitat and collected adult individuals of different amphibian species (orders Anura, Gymnophiona) by hand.

### 2.2 Critical thermal maximum and warming tolerance

We subjected individuals to Hutchison's dynamic method (Lutterschmidt & Hutchison, 1997), through a constant and gradual increase in temperature until reaching the Critical thermal maximum (CT_Max_) (see Taylor et al., 2020 for a review on thermal tolerance methods). We acclimated individuals for 72 hr at a room temperature of 25°C prior to assessing CT_Max_, following a 12‐hr photoperiod of light and dark regimes. We conducted the experiments in a specimen‐size experimental chamber with a 5‐mm high dechlorinated water layer (controlling the risk of desiccation), covered with a net to prevent individuals from escaping. We placed the experimental chambers inside a water bath to create a homogeneous heating system for experimental trials.

We started the bath's temperature at 25°C and gradually increased it by 0.25°C min^−1^ (Gutiérrez‐Pesquera et al., 2016). We used the “belly‐up” condition (Brattstrom, 1968; Taylor et al., 2020) to diagnose CT_Max_ for specimens by the lack of response to periodically employed stimuli (5 touches per min with a glass rod). Once the specimens were flipped over, exposing their venters, but remained immobile (not returning to normal position in 10 s), we considered the temperature of the chamber to have reached the CT_Max_ and the gradual warming was stopped. Subsequently, the chamber bottom temperature was measured using a contact thermometer (Miller & Weber, Inc.; 0.1°C accuracy). The specimens were then placed in a 23–25°C water container for cooling. We monitored all individuals for 24 hr, to ensure the lethal temperature was not reached during experiments, and only data from those who survived this period were included in the analyses. The CT_Max_ for each species was defined as the average of its sampled individuals. For each specimen, we measured snout–vent length (SVL) and head width (HW) with a 1‐mm precision caliper after the experimental protocol (including the observation time) in order to minimize the influence of handling in their survivorship. We also weigh (W) each individual just before experimental procedures using a 0.01‐g precision scale.

We obtained microclimate data (average temperature—*T*_Mean_; maximum temperature—*T*_Max_) by installing temperature data loggers (HOBO Pendant Temp/Light, UA‐002–64) in an area of regenerating Atlantic Forest remnants (secondary forest) during the three‐month (November—February) period with highest annual temperatures in the studied region. We installed a data logger in each microhabitat category used by amphibians (see section below). The warming tolerance (WT) was calculated from the difference between the CT_Max_ of each species and the maximum temperature of the microhabitat they use (sensu Deutsch et al., 2008), and we projected the future vulnerability by using the most recent International Panel on Climate Change (IPCC) indices for more and less acute warming scenarios (RCP 8.5–4.8°C; RCP 4.5–2.6°C, respectively) available on WorldClim database (worldclim.org). CT_Max_ and warming tolerance were used to access the thermal vulnerability of species for both scenarios. The Animal Research Ethics Committee of Santa Cruz State University authorized all experiments (Protocol No. 012/15), while collection license 13,708 was issued by SISBio/ICMBio.

### 2.3 Groupings and statistical analyses

We analyzed how ecological features of species covariate with CT_Max_ through a perspective of macrohabitat use (forest—Fo; generalist—Ge; and open areas—Op) and according to the site of larval development (lentic; lotic; marsupial; and terrestrial). To analyze possible trends in WT, we divided the species into groups by microhabitat (i.e., arboreal, cryptozoic, fossorial, and terrestrial). We based the functional group allocation of each individual species (see Table S2), following the ecological aspects available in Haddad et al. (2013).

Within functional groups, CT_Max_ was calculated from the average CT_Max_ of all individuals of the species assigned to each group. We compared our data on CT_Max_ of the adult's specimens graphically with those available in the literature for their respective conspecific tadpoles looking for ontogenetic tendencies on thermal tolerance (Supplementary Material in Gutiérrez‐Pesquera et al., 2016). We assessed differences in CT_Max_ and warming tolerance between groups by the nonparametric Kruskal–Wallis and the Dunn post hoc tests, with a significance level of 0.05.

We constructed radar charts to evaluate the thermal vulnerability of species given ecological covariates (e.g., macrohabitat, site of larval development, and microhabitat). We used the mean values of CT_Max_ obtained for each category (e.g., forest, generalist, and open areas) within the ecological covariates as a categorization parameter on a scale ranging from 1 to 3, where the value 1 was given to categories with the lowest average CT_Max_ within each covariate and the value 3 was given to those with the highest average CT_Max_.

### 2.4 Phylogenetic comparative methods

**FIGURE 1:**
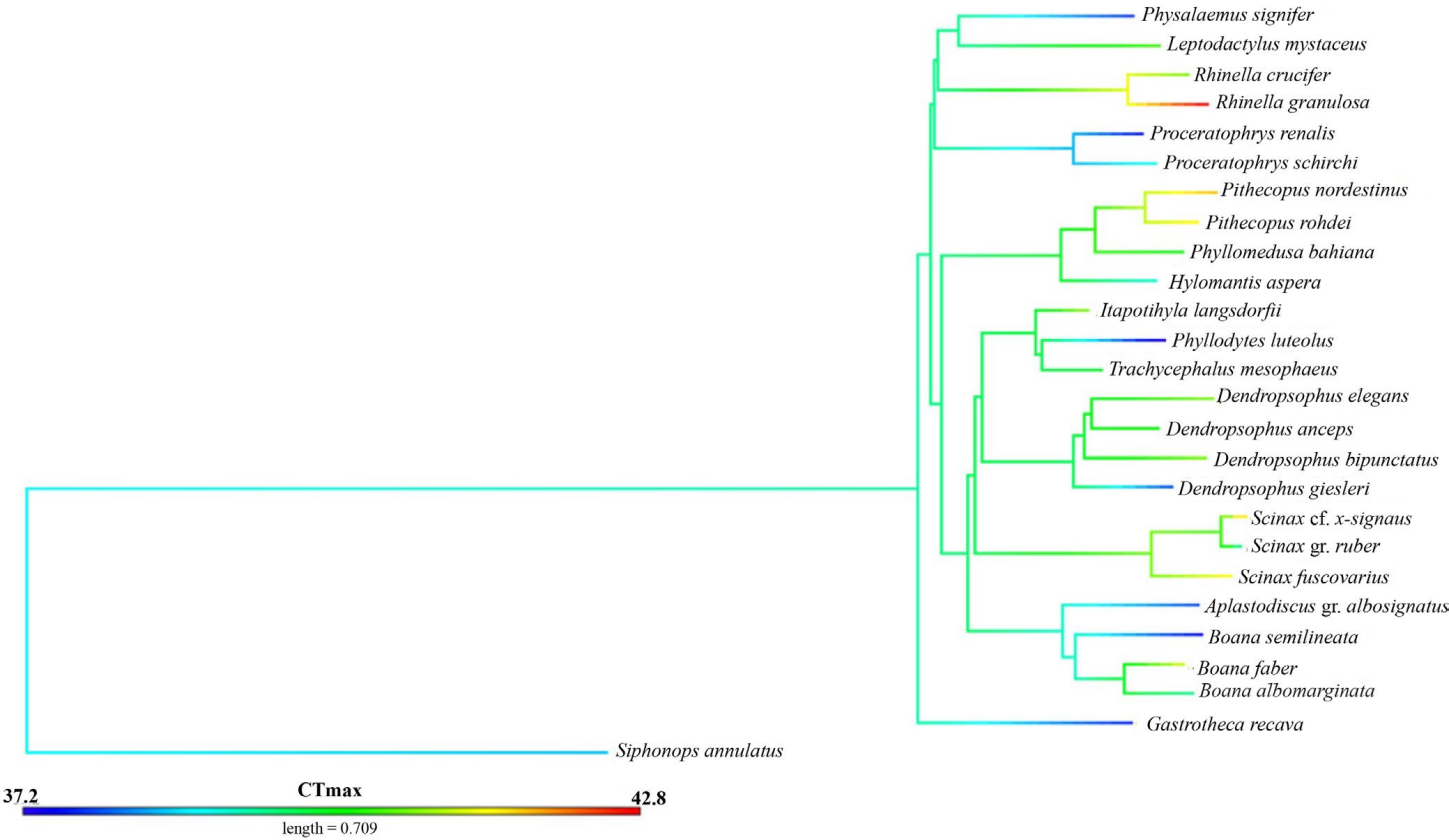
Phylogenetic tree including the 26 species studied (sensu Pyron & Wiens, 2011). Branch colors denote critical thermal maximum values for species. Color's gradient reaching from blue (lower CT_Max_) to red (higher CT_Max_)

Comparative methods that take phylogenetic information under consideration are broadly used when data on several species violate assumptions of statistical independence. Phylogenetic generalized least squares (PGLS) models use phylogenies to make estimates on expected covariance among species data (Garamszegi, 2014). We performed regression analysis by PGLS in a Brownian evolutionary motion model, using the CAPER package (Orme et al., 2013) in the statistical Program R (R Core Team, 2020) to test the effects of allometric (SVL, HL, and W), ecological (macrohabitat and microhabitat), and microclimatic (mean and maximum temperature—*T*_Mean_ and *T*_Max_) variables on CT_Max_. To account for phylogeny in analyses, we used the topology of Pyron and Wiens (2011), restricting the phylogeny to 23 species common with our sampling (Figure 1). We admitted the position of the nearest taxon, similar to Gutiérrez‐Pesquera et al. (2016), for three taxa (*Aplastodiscus* gr. *albosignatus*; *Scinax* gr. *ruber*; and *Scinax* cf. *x‐signatus*) that were not included in the phylogeny of Pyron and Wiens (2011). We used the lambda value to verify whether the covariance between the variables used follows the evolutionary pattern predicted by a Brownian motion model (where λ = 1) (Garamszegi, 2014). We ranked and selected among competing models using Akaike's information criteria (AIC). The model AIC weight value (wi) was used to evaluate the relative likelihood of model support (Burnham & Anderson, 2002). Analysis was conducted in R (R Core Team, 2020).

## 3 RESULTS

### 3.1 Environment and CT_Max_

We evaluated the environmental influences of CT_Max_ of 47 species (Table S1). CT_Max_ differed among species’ macrohabitat groups (*H* = 26.77, *df* =2, *p* < 0.01, *n* = 308) (Tables S1 and S2). CT_Max_ values of generalist species are intermediate between species that inhabit open environments (that reach higher values) and forest specialists (lower values) (Table 1). Pairwise statistics among groups of each covariate (macrohabitat, microhabitat, and site of larval development) are provided in Table 2.

**TABLE 1:** Variation of critical thermal maximum (CT_Max_) and warming tolerance (WT) by ecological covariates. Means and their respective standard deviations (mean ± *SD*), Sample size (*N*), Lower and upper ranges of the 95% confidence intervals (lower 95 and upper 95, respectively), The Kruskal–Wallis chi‐squared index (*H*) and analyses p‐value (*p*)

Critical thermal maximum						
	Mean ± *SD*	*N*	Lower 95	Upper 95	*H*	*p*
Habitat					26.8	<0.01
Forest	38.6 ± 1.7	192	38.3	38.8		
Generalist	39.5 ± 2.8	80	38.7	40.0		
Open Habitats	40.3 ± 1.5	36	39.8	41.0		
Larval development					27.7	<0.01
Lentic	39.3 ± 2.1	259	39.1	39.7		
Lotic	37.5 ± 0.9	31	36.9	37.7		
Marsupial	37.9 ± 0.8	4	36.6	39.1		
Terrestrial	38.6 ± 0.6	9	38.1	39.0		

Warming tolerance						
	Mean ± *SD*	*N*	Lower 95	Upper 95	*H*	*p*
Microhabitat					65.3	<0.01
Arboreal	7.0 ± 2.0	214	6.7	7.25		
Terrestrial	6.0 ± 1.8	27	5.27	6.71		
Cryptozoic	9.9 ± 1.3	34	9.5	10.4		
Fossorial	13.7 ± 0.0	9	13.2	14.1		

**TABLE 2:** Pairwise differences (Dunn's post hoc test) between covariates groups. Bold *p*‐values denote significant differences in the CT_Max_ of compared groups

Pairwise groups	*Z*‐test statistic	*p*‐value	Covariate
Forest – generalist	−3.06	**<0.01**	Macrohabitat
Forest – open areas	−4.73	**<0.01**	Macrohabitat
Generalist – open areas	−2.26	**0.01**	Macrohabitat
Lentic – lotic	5.12	**<0.01**	Site of larval development
Lotic – marsupial	−0.52	0.3	Site of larval development
Lentic – marsupial	1.38	0.08	Site of larval development
Marsupial – terrestrial	−0.74	0.22	Site of larval development
Terrestrial – lotic	−1.91	**0.03**	Site of larval development
Terrestrial – lentic	0.73	0.23	Site of larval development
Arboreal – cryptozoic	−6.86	**<0.01**	Microhabitat
Arboreal – fossorial	−5.42	**<0.01**	Microhabitat
Arboreal – terrestrial	1.82	**0.03**	Microhabitat
Cryptozoic – fossorial	−1.54	0.06	Microhabitat
Cryptozoic – terrestrial	6.35	**<0.01**	Microhabitat
Fossorial – terrestrial	5.76	**<0.01**	Microhabitat

CT_Max_ values between groups with different larval development sites were also different (*H* = 27.7, *df* = 3, *p* = 0.00, *n* = 303) (Tables S1 and S2). Species with larval development in lotic habitats had lower CT_Max_ values than species with larvae developing in lentic habitats (*p* < 0.01; Table 2). Our only marsupial frog species had lower CT_Max_ than the other groups with exception of the lotic species (Table 1). We sampled a single species of Caecilian (*Siphonops annulatus*, the only species in the terrestrial group). Its CT_Max_ was higher than that of marsupial anurans and species with lotic development and lower than that of lentic species. Our results regarding the terrestrial and marsupial group must be interpreted with caution, once in our sampling we were only able to collect individuals of only one species for each of these groups.

**FIGURE 2:**
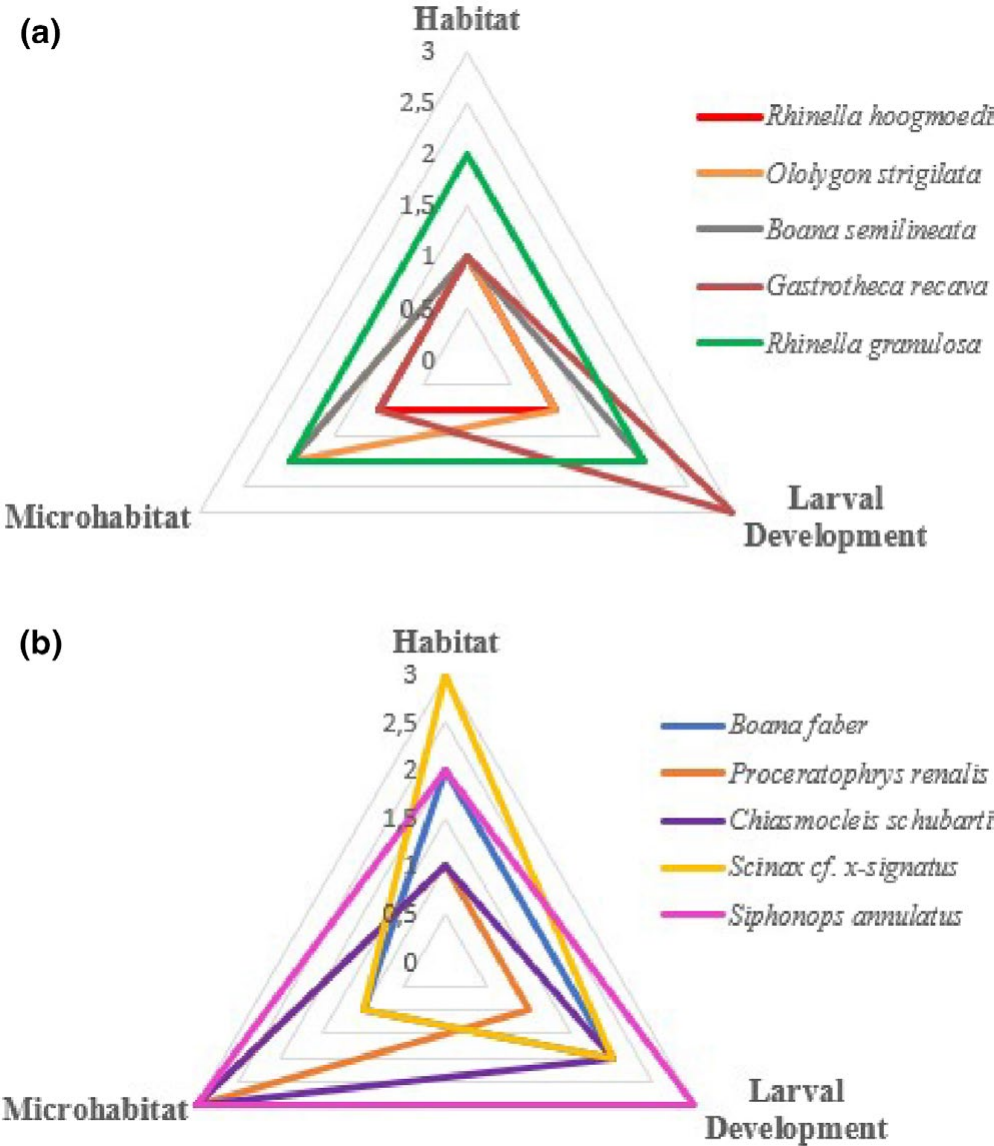
Radar charts showing ten patterns found for vulnerability according to ecological variables of macrohabitat, microhabitat, and larval development site. Each chart (a and b) is composed by five representative species for the general ecological patterns studied in the current study. Ecological categories were classified (1, 2, or 3) by CT_Max_ (ºC) values (1—less tolerant species; 3—more tolerant species) according to the results of ecological covariates in the group analyses. Charts with smaller areas denote greater thermal vulnerability than those with larger areas

We rank‐scaled our ecological covariates according to thermal tolerance within radar graphs using each covariate as one axis and recovered ten general patterns for our sampled species. Eight patterns had at least one covariate limiting their tolerance and increasing the vulnerability (value 1 in chart scale) (Figure 2). The most vulnerable pattern is of terrestrial species, with lotic larval development, that inhabit forests. In contrast, species that combine fossorial adult microhabitat with lentic larval development, and are habitat generalists, presented the most tolerant profile.

### 3.2 Warming tolerance

**FIGURE 3:**
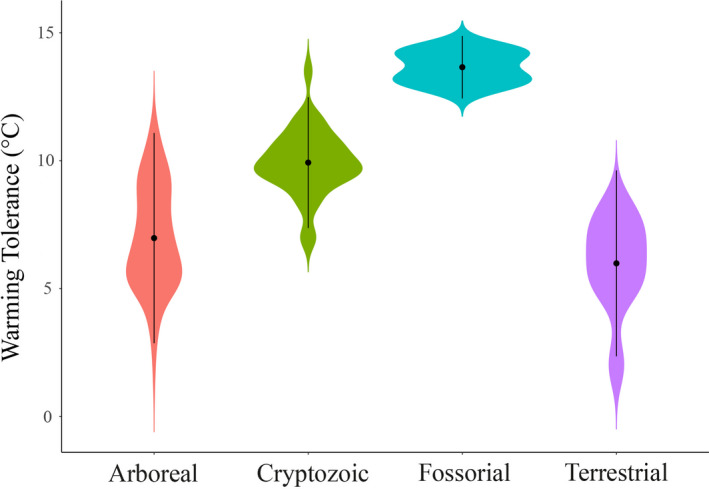
Effect of microhabitat on the warming tolerance of amphibian species from the Atlantic Forest of southern Bahia. Vertical bars denote 95% confidence intervals around the mean (point) of each functional group

The WT of the 47 studied amphibian species differed according to their microhabitats (*H* = 65.322, *df* = 3, *p* = 0.001, *n* = 284). Fossorial and cryptozoic species had higher warming tolerances than arboreal and terrestrial species (Figure 3). All functional groups showed values of warming tolerance different from each other (*p* < 0.05, Table 2), with the exception of cryptozoic and fossorial (z = −1.54, *p* = 0.06) (Table 1). Predicted climate change scenario of 2.6°C (RCP4.5) will raise thermal conditions (microhabitat maximum temperate) above the CT_Max_ of one species (*Rhinella hoogmoedi*) and near the thermal maximum of another one (*Dendropsophus haddadi*). Another predicted warming scenario (of 4.8°C) would increase the microhabitat maximum temperature above the CT_Max_ of three species (both previously mentioned and *Bokermannohyla capra*). This same scenario would drive nine additional species to experience thermal conditions near to their CT_Max_ (Table 3).

**TABLE 3:** Species most sensitive to temperature increase in view of more and less pessimistic scenarios proposed by the IPCC (2014) (RCP8.5 and RCP4.5, respectively). Values in the table refer to the maximum critical temperature (CT_Max_), species microhabitat, maximum critical temperature of microhabitats (*T*_Max_), current heating tolerance (WT), and those based on warming projections of IPCC (2014) (WT_RCP4.5_ and WT_RCP8.5_, respectively)

	CT_Max_ (°C)	Microhabitat	*T*_Max_ (°C)	WT	WT_RCP4.5_	WT_RCP8.5_
*Rhinella hoogmoedi*	37.5	Ter	35.4	2.1	−0.5	−2.7
*Dendropsophus haddadi*	35.8	Arb	31.9	3.9	1.3	−0.9
*Bokermannohyla capra*	36.5	Arb	31.9	4.6	2.0	−0.2
*Ololygon melanodactyla*	36.8	Arb	31.9	4.9	2.3	0.1
*Phyllodytes luteolus*	37.2	Arb	31.9	5.3	2.7	0.5
*Ololygon strigilata*	37.2	Arb	31.9	5.3	2.7	0.5
*Rhinella crucifer*	40.8	Ter	35.4	5.4	2.8	0.6
*Boana pombali*	37.3	Arb	31.9	5.4	2.8	0.6
*Boana semilineata*	37.5	Arb	31.9	5.6	3.0	0.8
*Aplastodiscus ibirapitanga*	37.6	Arb	31.9	5.7	3.1	0.9
*Gastrotheca recava*	37.8	Arb	31.9	5.9	3.3	1
*Dendropsophus giesleri*	37.9	Arb	31.9	6.0	3.4	1.2

### 3.3 PGLS model selection

The best‐supported model (AIC = 886, Wi = 0.63) indicated that amphibian CT_Max_ is explained by adult habitat and larval development site (Table 4). We did not observe a phylogenetic signal on CT_Max_ within the three best models.

**TABLE 4:** PGLS models. Italicized models are the five best‐selected models discussed on our results. Covariance adjustment parameter to the Brownian evolutionary model (λ), Akaike's information criterion value (AIC), Akaike's weight (wi), Snout–vent length (SVL), Head width (HW), Site of larval development (LDS), Average microhabitat temperature (*T*_Mean_), Maximum microhabitat temperature (*T*_Max_) and Weight (W)

Model	Formulation	λ	AIC	wi
*m12*	*CT_Max_ ~ Habitat + LDS*	*0*	*887*	*0.63*
*m9*	*CT_Max_ ~ HW/SVL + Habitat + LDS*	*0*	*923*	*0.10*
*m10*	*CT_Max_ ~ W/SVL + Habitat + LDS*	*0*	*924*	*0.10*
*m5*	*CT_Max_ ~ T_mean_ + T_max_ + Habitat + LDS*	*0*	*926*	*0.01*
*m7*	*CT_Max_ ~ HW/SVL + T_mean_ + T_max_ + Habitat + LDS*	*0*	*961*	*0.01*
m6	CT_Max_ ~ SVL * HW * W + *T*_mean_ + *T*_max_ + Habitat + LDS	1	964	0.01
m8	CT_Max_ ~ W/SVL + *T*_mean_ + *T*_max_ + Habitat + LDS	0	964	0.01
m11	CT_Max_ ~ SVL * HW * W + Habitat + LDS	0.75	964	0.01
m13	CT_Max_ ~ *T*_mean_ + *T*_max_	0	981	0.00
m1	CT_Max_ ~ W/SVL + *T*_max_	0	994	0.00
m3	CT_Max_ ~ SVL * HW * W + *T*_max_	0	10	0.00
m4	CT_Max_ ~ SVL * HW * W + *T*_mean_	0	101	0.00
m2	CT_Max_ ~ W/SVL + *T*_mean_	0	103	0.00

## 4 DISCUSSION

Literature suggests that available macro‐ and microhabitats play an important role in thermoregulation and ecophysiology of forest‐associated tropical amphibians (Nowakowski et al., 2018; Scheffers et al., 2013, 2014). Our results corroborate this notion because values of adult amphibian CT_Max_ of forest species were significantly lower than those from generalist or open environment species. Canopy protection and the availability of thermal refuges seem to be crucial to avoid overheating of forest species due to their low heat tolerance (Scheffers et al., 2014). Thus, the species of amphibians that inhabit forests will depend on even less macro‐ and microhabitats available in a global context marked by habitat loss (Stuart et al., 2004) and the exposure of thermal refuges to rising temperatures (Ficetola et al., 2015).

Among the three best models, there was no association between the CT_Max_ and the *T*_Mean_ and *T*_Max_ of the microhabitat. Literature suggests that the CT_Max_ of aquatic ectothermic organisms (e.g., crustaceans, fish, and aquatic insects) is more influenced by average and maximum environment temperatures because terrestrial ectothermic organisms are able to use habitat heterogeneity to exploit different microenvironments in order to maintain their body temperatures independent of thermal averages from the air (Bogert, 1949; Gunderson & Stillman, 2015; Kearney et al., 2009; Stevenson, 1985; Sunday et al., 2011).

In a study conducted in Costa Rica, Frishkoff et al. (2015) found an association between the occurrences of lentic larval development species (i.e., puddles) with deforested areas, while those with lotic larval development (i.e., streams) or direct development seemed to prefer forests. In our study, species that reproduce in ponds tolerated higher temperatures than lotic species, suggesting that lentic species that can eventually use lotic water bodies for reproduction (e.g., some species from genus *Boana*, *Aplastodiscus*, and *Rhinella*) are better competitors than lotic breeders in warmer environments (Haddad et al., 2013). Due to the low sample size of marsupial and direct development species, we were unable to characterize variation in CT_Max_ with confidence for these groups. However, species that do not depend on water bodies for their development depend on the humidity of the forest to prevent desiccation of eggs (Frishkoff et al., 2015; Scheffers et al., 2013).

The Atlantic Forest faces a historical crisis of deforestation and fragmentation (Moura et al., 2018; SOS Mata Atlântica, 2017; Wanger et al., 2020). As degradation usually implies increased edge effect on forest matrices (Kapos, 1989), and consequently a higher incidence of light, forest specialist taxa are exposed to microclimatic modifications of their thermal refuges (Nowakowski et al., 2018; Tuff et al., 2016). Once exposed to anomalous and stressful thermal conditions, forest specialist species amphibians in southern Bahia face the risk of isolation and population disturbance from forest fragmentation, which associated with possible thermal stress could lead to local extinction events (Becker et al., 2007; Tuff et al., 2016). In a region with high amphibian richness and endemism rates (Carnaval et al., 2009; Dias et al., 2014; Mira‐Mendes et al., 2018; Vasconcelos et al., 2014), such a scenario raises an alarm concerning the extinction risk of endemic species.

Amphibians are particularly sensitive to habitat loss and fragmentation (Becker et al., 2007), and regenerating environments (secondary forests) usually present lower species richness when compared to pristine environments (Thompson & Donelly, 2018). According to Schneider‐Maunoury et al. (2016), the consequences of edge effects (resulting from degradation of forest matrices) on amphibians are related to variables of the general biology of species such as body size and habitat specialization. Our results show a converging panorama when we recover that the CT_Max_, an ecophysiological variable, is strongly influenced by adult and larval habitat, as well as when we highlight considerable differences between the CT_Max_ of forest species, generalists, and those inhabiting open environments.

In the present study, the most tolerant species were those with cryptozoic or fossorial microhabitat. Nevertheless, other categories (arboreal and terrestrial) maintained their WT within tolerable physiological limits, according to current thermal signatures. We found 2–12 species that will be at risk of extinction given likely climate change scenarios in the Atlantic Forest considering the projections of temperature increase between 2.6 and 4.8°C (RCP 4.5 and RCP 8.5, respectively—IPCC, 2014). Terrestrial and arboreal species were more sensitive (lower WT) than cryptozoic and fossorial ones. Given that microhabitat warming can also be a consequence of forest cover loss, cryptozoic and fossorial species are also endangered by the increase in temperature and risk of desiccation (Kapos, 1989; Nowakowski et al., 2018; Tuff et al., 2016).

Even though our results point to habitat as the variable that best explains CT_Max_, the influence of the environment is not restricted to the upper tolerance limits. Diversity within families of anuran amphibians is mainly explained by species microhabitat, and to a lesser extent by the thermal niche they occupy (Moen & Wiens, 2017). However, several studies have recovered phylogenetic signals in niche thermal dimensions for various groups, including amphibians (e.g., Gutiérrez‐Pesquera et al., 2016; Hof et al., 2010; Olalla‐Tárraga et al., 2011). Our results agree with Moen and Wiens (2017) because CT_Max_ values do not result from an evolutionary process predicted by the Brownian motion model (best‐adjusted models, λ = 0). Instead, we recovered a strong influence of the adult and tadpole habitat for the adult CT_Max_ within the studied species, thus indicating that the occupation of the environment is an important factor to explain the heating tolerance of amphibian species. Previous research also showed an ecological pattern for the thermal limits in some Neotropical anurans from the superfamily Brachycephaloidea where physiological traits were positively correlated with the altitudinal distribution of the evaluated taxa (von May et al., 2017).

**FIGURE 4:**
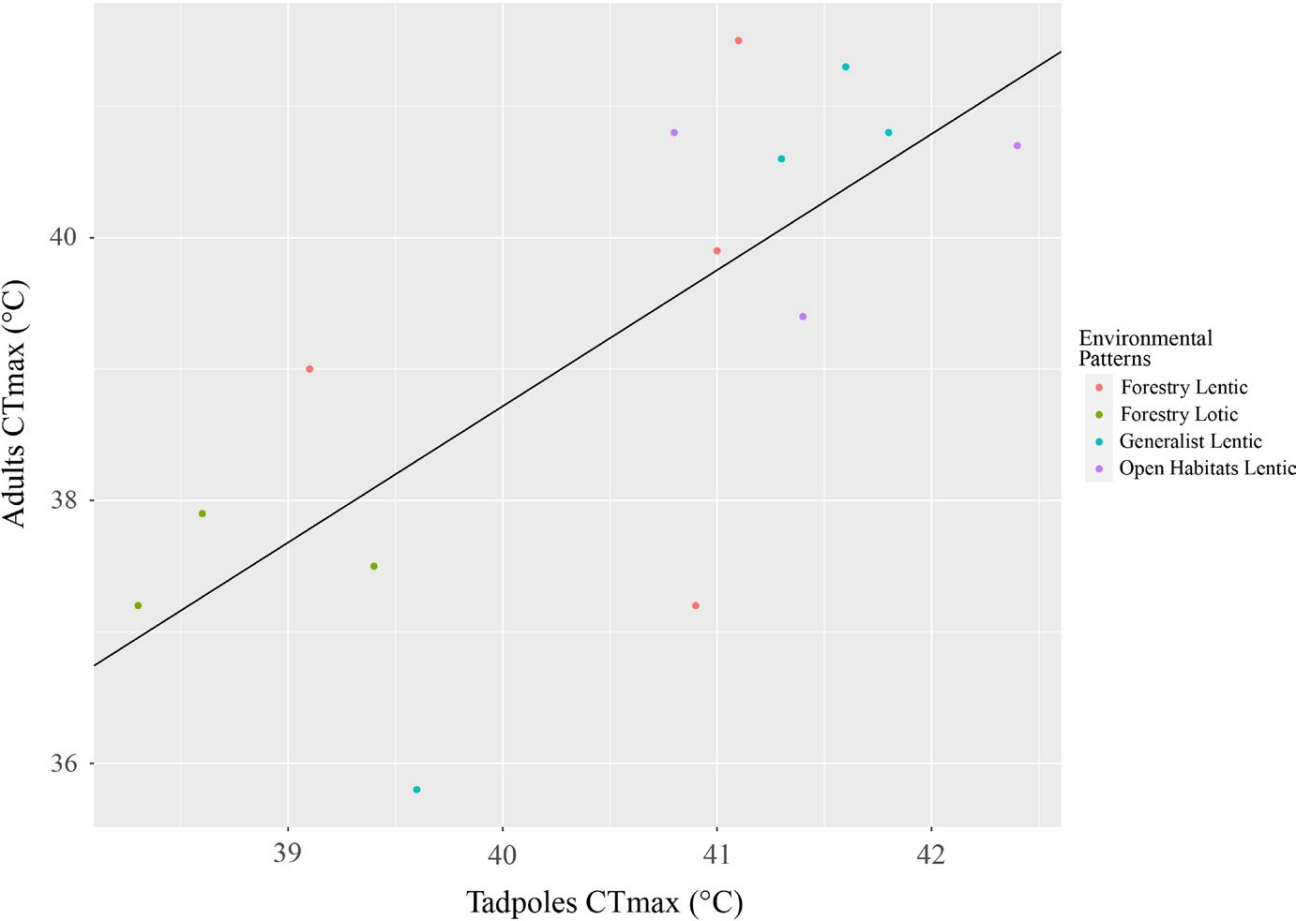
Distribution of CT_Max_ data between adults and tadpoles of 14 species of anurans from southern Bahia. Data of tadpoles were obtained from Gutiérrez‐Pesquera et al. (2016)

Given that our analysis did not recover any phylogenetic signal, to what extent does the adaptive potential of the analyzed Amphibia groups influence survival in a warming landscape? According to Moritz et al. (2012), although no significant variation was found in CT_Max_ between peripheral and central lineages of the same species, there are differences among upper thermal limits between edge species and those within forest. Hypothetically, populations at the periphery of fragments may present genotypes that give them some resilience to future warming scenarios (see Moritz et al., 2012). However, phenotypic plasticity seems to have little ability to buffer harmful effects in a progressive context of rising global temperatures (Bellard et al., 2012). Terrestrial ectotherms have low acclimation capacity when it comes to temperature rises, and therefore are less tolerant to temperature changes than aquatic ectotherms (Gunderson & Stillman, 2015). We observed that differences between the adult CT_Max_ (this study) and tadpoles of 14 species (Gutiérrez‐Pesquera et al., 2016) confirm the previously presented pattern—tadpoles (aquatic environment) tend to present higher maximal CT_Max_ than adults (environment specific) (Figure 4).

For tadpoles, Tejedo et al. (2012) point out that the ability to adapt to temperature increase is linked to the thermal environments experienced by lineages. Thus, generalist species (tolerating more variable thermal environments) would be better adapted to cope with temperature rises than thermally specialized taxa (e.g., forest and open‐air species) (Tejedo et al., 2012). Although our data for adult amphibians also indicate such a pattern, it is still necessary to investigate whether larval forms exhibit similar behavior for thermal tolerance as a function of aquatic microhabitat. Although preliminary, we note that the tolerance patterns between larvae and adults signaled here point to a thermal separation between the adult and larval life stages (Becker et al., 2007).

By combining the data available from Gutiérrez‐Pesquera et al. (2016) for species with low plasticity in tolerance limits (Gunderson & Stillman, 2015; Tejedo et al., 2012), progressive reduction of forest patches (SOS Mata Atlântica), and the projections of temperature increase (IPCC, 2014), we point to threat scenarios close to those projected by previous studies (Ceballos et al., 2017). The consequences for such scenarios pass through population reductions (Becker et al., 2007) that can lead to loss of genetic diversity for populations (Ceballos et al., 2017; Moritz et al., 2012) and species (Carnaval et al., 2009), as well as extinction events (Alroy, 2015; Ceballos et al., 2017).

Overall, our results reinforce the general understanding that global warming is certainly a threat to biodiversity but produces different pressures according to the natural history of each species. Deforestation and homogenization of microhabitats can potentiate the effects of global warming and need to be evaluated synergistically. Interpretation of the results through the “Radar” charts (Figure 2) shows that even species classified as Least Concern or Data Deficient by IUCN are facing thermal risk. Overlapping graphical patterns (e.g., radar charts) from a scale established by the CT_Max_ averages denoted the specific influence of macro‐ and microhabitat as a vulnerability predictor of southern Bahia amphibians. Even in radar patterns with the same area, different ecological aspects lead to classify species as vulnerable to thermal stress. The results obtained from the model selection confirmed the same trend. Therefore, the use of graphic components based on ecological variables, as well as the application of rigorously established scales, proved to be an efficient risk/vulnerability assessment strategy for the species studied here. The same approach may be used to estimate the susceptibility of other species, or even groups, to anthropogenic impacts.

The restrictions imposed by the thermal niches must be taken into account for conservation actions (Araújo et al., 2013; Damasceno et al., 2014). Thermal limit data are only available for about one tenth of Atlantic Forest amphibians—ca. 65 species. (Gutiérrez‐Pesquera et al., 2016; Simon et al., 2015; Tejedo et al., 2012; present study). In addition to CT_Max_, other ecophysiological variables (e.g., voluntary limits) could improve vulnerability projections for Atlantic Forest species if implemented in mechanistic niche models (Taylor et al., 2020). Our research shows that natural history plays a key role in thermal tolerance and thermal vulnerability in amphibians of the Brazilian Atlantic Forest and may be a proxy for thermal niche. Considering the low latitudinal variation of CT_Max_ and conservationism in the upper thermal limits (Araújo et al., 2013), our results can be used for extrapolations within this biome.

## CONFLICT OF INTEREST

None declared.

## AUTHOR CONTRIBUTIONS

**Leildo Machado Carilo Filho:** Conceptualization (lead); Data curation (lead); Formal analysis (lead); Investigation (equal); Methodology (lead); Validation (lead); Visualization (lead); Writing – original draft (lead); Writing – review and editing (lead). **Bruno Teixeira de Carvalho:** Conceptualization (equal); Data curation (equal); Investigation (equal); Methodology (equal). **Bruna Kelly Alves de Azevedo:** Data curation (equal); Methodology (equal). **Luis Miguel Gutiérrez‐Pesquera:** Conceptualization (equal); Data curation (equal); Methodology (equal); Validation (equal); Writing – review and editing (equal). **Caio Vinícius Mira‐Mendes:** Conceptualization (equal); Methodology (equal); Writing – original draft (equal); Writing – review and editing (equal). **Mirco Solé:** Conceptualization (equal); Funding acquisition (equal); Methodology (equal); Resources (equal); Supervision (equal); Writing – original draft (equal); Writing – review and editing (equal). **Victor Goyannes Dill Orrico:** Conceptualization (equal); Formal analysis (equal); Funding acquisition (equal); Methodology (equal); Project administration (equal); Resources (equal); Supervision (equal); Writing – original draft (equal); Writing – review and editing (equal).

## Supporting information

Tables S1 and S2

## Data Availability

Data sources used for statistical group analyses, species traits, and most sensitive taxa are provided in the Supporting Information. All files mentioned above are available online at https://doi.org/10.5061/dryad.ttdz08kzp.
