# Supplementary material for: Natural history predicts patterns of thermal vulnerability in amphibians from the Atlantic Rainforest of Brazil
Source: Ecol Evol. 2021 Nov 19;11(23):16462–72. doi: 10.1002/ece3.7961 (PMC8668723; doi:10.1002/ece3.7961)
Supplement: Supplementary file 1 — Tables S1 and S2 [file ECE3-11-16462-s001.docx]

**Natural History Predicts Patterns of Thermal Vulnerability in Amphibians from the Atlantic Rainforest of Brazil**

Leildo M. Carilo Filho^1*^, Bruno T. de Carvalho^1^, Bruna K. A. Azevedo^2^, Luis M. Gutiérrez-Pesquera^4^, Caio V. Mira-Mendes^3^, Mirco Solé^1,5^, Victor G.D. Orrico^1^

**ONLINE RESOURCE - SUPPLEMENTARY MATERIAL**

**Table 1S** List of species used for thermal tolerance assessment; species were arranged in a crescent order of their warming tolerance values. Critical maximum temperature (CT_Max_), microhabitat (Haddad et al., 2013), maximum microhabitat temperature (T_Max_), current warming tolerance (WT) and number of individuals used to obtain CT_Max_ (N).

|  | CT_Max_ (°C) | Microhabitat | T_Max_ (°C) | WT | N |
| --- | --- | --- | --- | --- | --- |
| *Rhinella hoogmoedi* | 37.5 | Ter | 35.4 | 2.1 | 3 |
| *Dendropsophus haddadi* | 35.8 | Arb | 31.9 | 3.9 | 14 |
| *Bokermannohyla capra* | 36.5 | Arb | 31.9 | 4.6 | 1 |
| *Ololygon melanodactyla* | 36.8 | Arb | 31.9 | 4.9 | 14 |
| *Phyllodytes luteolus* | 37.2 | Arb | 31.9 | 5.3 | 23 |
| *Ololygon strigilata* | 37.2 | Arb | 31.9 | 5.3 | 10 |
| *Rhinella crucifer* | 40.8 | Ter | 35.4 | 5.4 | 11 |
| *Boana pombali* | 37.3 | Arb | 31.9 | 5.4 | 2 |
| *Boana semilineata* | 37.5 | Arb | 31.9 | 5.6 | 14 |
| *Aplastodiscus ibirapitanga* | 37.6 | Arb | 31.9 | 5.7 | 1 |
| *Gastrotheca recava* | 37.7 | Arb | 31.9 | 5.8 | 4 |
| *Dendropsophus giesleri* | 37.9 | Arb | 31.9 | 6 | 1 |
| *Ololygon argyreornata* | 38 | Arb | 31.9 | 6.1 | 13 |
| *Aplastodiscus sibilatus* | 38.2 | Arb | 31.9 | 6.3 | 1 |
| *Gabohyla pauloalvini* | 38.2 | Arb | 31.9 | 6.3 | 14 |
| *Boana atlantica* | 38.8 | Arb | 31.9 | 6.9 | 5 |
| *Hylomantis aspera* | 39 | Arb | 31.9 | 7.1 | 6 |
| *Dendropsophus nekronastes* | 39.1 | Arb | 31.9 | 7.2 | 5 |
| *Rhinella granulosa* | 42.8 | Ter | 35.4 | 7.4 | 13 |
| *Scinax* gr. *ruber* | 39.3 | Arb | 31.9 | 7.4 | 7 |
| *Boana albomarginata* | 39.4 | Arb | 31.9 | 7.5 | 7 |
| *Trachycephalus mesophaeus* | 39.9 | Arb | 31.9 | 8 | 2 |
| *Dendropsophus anceps* | 40.2 | Arb | 31.9 | 8.3 | 1 |
| *Phyllomedusa bahiana* | 40.2 | Arb | 31.9 | 8.3 | 11 |
| *Scinax juncae* | 40.4 | Arb | 31.9 | 8.5 | 10 |
| *Frostius erythrophthalmus* | 36.7 | Crip | 28.2 | 8.5 | 2 |
| *Sphaenorhynchus prasinus* | 40.6 | Arb | 31.9 | 8.7 | 3 |
| *Scinax eurydice* | 40.7 | Arb | 31.9 | 8.8 | 1 |
| *Dendropsophus bipunctatus* | 40.8 | Arb | 31.9 | 8.9 | 2 |
| *Dendropsophus elegans* | 40.8 | Arb | 31.9 | 8.9 | 1 |
| *Itapotihyla langsdorfii* | 40.9 | Arb | 31.9 | 9 | 4 |
| *Adenomera thomei* | 37.4 | Crip | 28.2 | 9.2 | 3 |
| *Boana faber* | 41.3 | Arb | 31.9 | 9.4 | 1 |
| *Proceratophrys renalis* | 37.6 | Crip | 28.2 | 9.4 | 9 |
| *Physalaemus signifer* | 37.8 | Crip | 28.2 | 9.6 | 6 |
| *Scinax* cf. *fuscovarius* | 41.5 | Arb | 31.9 | 9.6 | 1 |
| *Pithecopus rohdei* | 41.5 | Arb | 31.9 | 9.6 | 20 |
| *Chiasmocleis schubarti* | 37.9 | Crip | 28.2 | 9.7 | 6 |
| *Chiasmocleis gnoma* | 38 | Crip | 28.2 | 9.8 | 1 |
| *Pithecopus nordestinus* | 41.8 | Arb | 31.9 | 9.9 | 11 |
| *Scinax* cf. *x-signatus* | 41.9 | Arb | 31.9 | 10 | 11 |
| *Leptodactylus natalensis* | 38.5 | Crip | 28.2 | 10.3 | 1 |
| *Proceratophrys schirchi* | 38.9 | Crip | 28.2 | 10.7 | 2 |
| *Stereocyclops incrassatus* | 39.2 | Crip | 28.2 | 11 | 5 |
| *Leptodactylus mystaceus* | 40.5 | Crip | 28.2 | 12.3 | 2 |
| *Siphonops annulatus* | 38.5 | Fos | 24.9 | 13.6 | 9 |
| *Allobates olfersioides* | 35.8 | Reo | - | - | 3 |

**Table 2S**. List of species used for Radar graphs. Habitat (Fo- Forestry; Ge – Generalist; Op – Open Areas). Microhabitat (Arb – Arboreal; Ter – Terrestrial; Crip – Criptozoic; Fos - Fossorial). Larval Development Space (Lo – Lotic; Le- Lentic; Dir – Direct development). Radar patterns were obtained from ecological traits combinations of the evaluated species. Species without ecological data available were not included.

|  | Habitat | Microhabitat | LDS | Pattern |
| --- | --- | --- | --- | --- |
| *Rhinella hoogmoedi* | Fo | Ter | Lo | 2 |
| *Dendropsophus haddadi* | Ge | Arb | Le | 3 |
| *Bokermannohyla capra* | Fo | Arb | Lo | 5 |
| *Ololygon melanodactyla* | Ge | Arb | Le | 3 |
| *Phyllodytes luteolus* | Fo | Arb | Le | 7 |
| *Ololygon strigilata* | Fo | Arb | Lo | 5 |
| *Rhinella crucifer* | Ge | Ter | Le | 8 |
| *Boana pombali* | Fo | Arb | Le | 7 |
| *Boana semilineata* | Fo | Arb | Le | 7 |
| *Aplastodiscus ibirapitanga* | Fo | Arb | Lo | 5 |
| *Dendropsophus giesleri* | Op | Arb | Le | 1 |
| *Ololygon argyreornata* | Fo | Arb | Le | 7 |
| *Aplastodiscus sibilatus* | Fo | Arb | Lo | 5 |
| *Gabohyla pauloalvini* | Fo | Arb | Le | 7 |
| *Boana atlantica* | Op | Arb | Le | 1 |
| *Hylomantis aspera* | Fo | Arb | Le | 7 |
| *Dendropsophus nekronastes* | Op | Arb | Le | 1 |
| *Rhinella granulosa* | Ge | Ter | Le | 8 |
| *Scinax* gr. *ruber* | Fo | Arb | Le | 7 |
| *Boana albomarginata* | Op | Arb | Le | 1 |
| *Trachycephalus mesophaeus* | Fo | Arb | Le | 7 |
| *Dendropsophus anceps* | Op | Arb | Le | 1 |
| *Phyllomedusa bahiana* | Fo | Arb | Le | 7 |
| *Scinax juncae* | Fo | Arb | Le | 7 |
| *Sphaenorhynchus prasinus* | Ge | Arb | Le | 3 |
| *Scinax eurydice* | Op | Arb | Le | 1 |
| *Dendropsophus bipunctatus* | Op | Arb | Le | 1 |
| *Dendropsophus elegans* | Op | Arb | Le | 1 |
| *Itapotihyla langsdorfii* | Fo | Arb | Le | 7 |
| *Boana faber* | Ge | Arb | Le | 3 |
| *Proceratophrys renalis* | Fo | Crip | Lo | 4 |
| *Physalaemus signifer* | Fo | Crip | Le | 6 |
| *Scinax* cf. *fuscovarius* | Op | Arb | Le | 1 |
| *Pithecopus rohdei* | Fo | Arb | Le | 7 |
| *Chiasmocleis schubarti* | Fo | Crip | Le | 6 |
| *Chiasmocleis gnoma* | Fo | Crip | Le | 6 |
| *Pithecopus nordestinus* | Ge | Arb | Le | 3 |
| *Scinax* cf. *x-signatus* | Op | Arb | Le | 1 |
| *Leptodactylus natalensis* | Fo | Crip | Le | 6 |
| *Proceratophrys schirchi* | Fo | Crip | Lo | 4 |
| *Stereocyclops incrassatus* | Fo | Crip | Le | 6 |
| *Leptodactylus mystaceus* | Fo | Crip | Le | 6 |
| *Siphonops annulatus* | Ge | Fos | Dir | 9 |
| *Gastrotheca recava* | Fo | Arb | Dir | 10 |
